# Can MRI T_1_ be used to detect early changes in 5xFAD Alzheimer’s mouse brain?

**DOI:** 10.1007/s10334-016-0593-9

**Published:** 2016-10-26

**Authors:** Nicholas G. Spencer, David P. Lovell, Kay Elderfield, Brian Austen, Franklyn A. Howe

**Affiliations:** 1 Institute for Infection and Immunity, St George’s, University of London, Cranmer Terrace, London, SW17 0RE UK; 2 Institute of Medical and Biomedical Education, St. George’s, University of London, Cranmer Terrace, London, SW17 0RE UK; 3 Leica Biosystems, Larch House, Woodlands Business Park, Breckland, Linford Wood, Milton Keynes, Buckinghamshire MK14 6FG UK; 4 Molecular and Clinical Sciences Institute, St George’s, University of London, Cranmer Terrace, London, SW17 0RE UK

**Keywords:** Alzheimer’s disease, Transgenic mice, Magnetic resonance imaging, Alzheimer beta-protein, Inflammation

## Abstract

**Objectives:**

In the present study, we have tested whether MRI T_1_ relaxation time is a sensitive marker to detect early stages of amyloidosis and gliosis in the young 5xFAD transgenic mouse, a well-established animal model for Alzheimer’s disease.

**Materials and methods:**

5xFAD and wild-type mice were imaged in a 4.7 T Varian horizontal bore MRI system to generate T_1_ quantitative maps using the spin-echo multi-slice sequence. Following immunostaining for glial fibrillary acidic protein, Iba-1, and amyloid-β, T_1_ and area fraction of staining were quantified in the posterior parietal and primary somatosensory cortex and corpus callosum.

**Results:**

In comparison with age-matched wild-type mice, we observed first signs of amyloidosis in 2.5-month-old 5xFAD mice, and development of gliosis in 5-month-old 5xFAD mice. In contrast, MRI T_1_ relaxation times of young, i.e., 2.5- and 5-month-old, 5xFAD mice were not significantly different to those of age-matched wild-type controls. Furthermore, although disease progression was detectable by increased amyloid-β load in the brain of 5-month-old 5xFAD mice compared with 2.5-month-old 5xFAD mice, MRI T_1_ relaxation time did not change.

**Conclusions:**

In summary, our data suggest that MRI T_1_ relaxation time is neither a sensitive measure of disease onset nor progression at early stages in the 5xFAD mouse transgenic mouse model.

## Introduction

Alzheimer’s disease (AD) is clinically characterised by the increasing decline of cognitive functions that eventually leaves the patient dependent on custodial care. Studies in sporadic and autosomal dominant AD support a sequence of pathological changes, namely increased brain amyloidosis, tau pathology, decreased glucose metabolism, neurodegeneration, and brain atrophy—with amyloidosis being the earliest component, starting years or even decades before clinical symptoms, i.e., cognitive decline, begin to show [1–3]. In addition, there is now good evidence for neuroinflammation in the brain of AD patients [4, 5]. Activated microglia and astrocytes appear to play key roles in regulating AD pathogenesis, while they can have both protective and detrimental effects [6–15].

Current diagnostic criteria used to identify AD in people with dementia rely on advanced neurological impairment—which is irreversible with current treatment options. To date, diagnostic markers that are sensitive to early disease stages are not available. Early biomarkers of AD can aid presymptomatic diagnosis, drug development by providing surrogate endpoints, treatment decisions, and disease monitoring [16]. Neuroimaging techniques using positron emission tomography (PET) exist to measure changes in glucose metabolism, currently the most common diagnostic criterion for AD [17], and also amyloid-β (Aβ) deposition, tau aggregation, and neuroinflammation. Magnetic resonance imaging (MRI) is also recognised as an important tool in AD diagnosis and has some advantages over PET in that it is cheaper and currently more widely available. MRI enables visualisation of the medial temporal lobe, in which atrophy is related to AD and its severity [18, 19]. Although MRI is already routinely used to monitor disease progression at late stages of AD, i.e., after the onset of cognitive decline, it remains unclear whether this technique can also be applied to identify early changes in the brain of AD patients before clinical symptoms become apparent.

In a cross-sectional analysis of 21 subjects with AD and 32 similarly aged healthy controls, AD was characterised by reduced quantitative T_1_ and T_2_ values [20]. In previous work by the same authors, decreased T_1_ values were found in patients with dementia with Lewy bodies compared with control subjects, highlighting the potential of T_1_ measures in general to detect changes caused by neurodegeneration [21]. Using 5xFAD transgenic mice, a well-recognised AD mouse model [22–24], we have previously shown that at the age of 11 months, 5xFAD mice have reduced MRI T_1_ relaxation times in the lower layers of the posterior parietal and primary somatosensory areas of the cortex and the corpus callosum compared with age-matched wild-type controls [25]. The cortex has a complex architecture that is made up of several layers, each defined by varying densities of myelin, neuronal cells, and receptors [26]. Aβ deposition also follows a layered pattern in the cortex [25, 27]; regional variations of tissue changes related to Aβ deposition, gliosis, and/or neuronal loss may not be captured in large regions of interest that include less affected areas of tissue. We previously found in the cortex of 11-month-old 5xFAD mice a pattern of T_1_ reductions that broadly correspond to the pattern of Aβ reduction [25]. This indicates that quantitative MRI can provide information about tissue changes in specific regions of the mouse brain provided regions of interest (ROI) are placed appropriately. Due to the higher spatial resolution allowed by quantitative-T_1_ MRI than diffusion tensor imaging and magnetisation transfer ratio MRI, quantitative T_1_ measurements have better chance of detecting changes in small regions of the brain. Here, we have extended these studies on young 5xFAD mice to test whether the determination of MRI T_1_ relaxation times is a sensitive method capable of identifying early changes in the AD mouse brain, such as the beginning of amyloidosis and gliosis.

## Materials and methods

### Transgenic mice

This study was carried out in strict accordance with the UK Home Office Animals Scientific Procedures Act 1986. The protocol was approved by the committee of the UK Home Office (PPL number: 70/7075). All efforts were made to minimise suffering. Mice were purchased from Jackson Laboratory (5xFAD strain name: B6SJL-Tg[APPSwFlLon, PSEN1*M146L*L286 V]6799Vas/Mmjax]; stock number: 006554; and non-carrying [wild-type] mice strain name: B6SJF1/J; stock number: 100012). A colony of transgenic and wild-type controls was produced by crossing hemizygous 5xFAD mice with wild-type mice. Mice were genotyped using polymerase chain reaction and gel electrophoresis. Eleven young mice [five wild-type and six 5xFAD (mean age = 2.6 months, SD = 0.16)] and ten older mice [five wild-type and five 5xFAD (mean age = 5.2 months, SD = 0.21)] were imaged. For simplicity, we describe these ages as 2.5 and 5 months throughout this report. A mixture of males and females were used in all groups. To compare Aβ load in young 5xFAD mice with that of old mice, we additionally quantified Aβ load in stained sections from 11-month-old mice that were previously obtained using the same methods of animal breeding and immunohistochemistry.

### In vivo MRI

In vivo MR images were acquired on a 4.7 T Varian horizontal bore MRI system with a mouse brain coil setup comprising a volume transmit coil and surface coil receiver (RAPID Biomedical GmBH, Würzburg-Rimpar, Germany). Mice were initially anaesthetised with isoflurane, consisting of an O_2_ mixture (2 L/min), administered in a sealed tank fitted to an isoflurane scavenger system. Subsequently, a 1:2:1 mixture of Hypnorm (0.315 μg/mL fentanyl citrate and 10 mg/mL fluanisone), sterile water, and Hypnovel (5 mg/mL midazolam) was administered intraperitoneally at 10 mg/kg bodyweight. Body temperature was maintained by a warming bed integrated into the imaging coil apparatus.

For each mouse, five images were acquired using the spin echo multi-slice sequence with repetition times (TR) of 250, 500, 1000, 2000, 4000 ms and 16, 8, 4, 2, 2 averages, respectively, to maintain a similar signal-to-noise ratio for each TR. Other imaging parameters remained constant for all images: echo time (TE) = 14 ms; field of view = 30 × 30 mm; matrix = 128 × 128; nine contiguous slices of 1 mm thickness with the most anterior of the nine slices positioned just behind the olfactory bulb; total acquisition time = 51.2 min. The range of TRs used was chosen to adequately quantify the range of T_1_ relaxation times in the mouse brain. Immediately after imaging, the mouse was culled using the Schedule 1 method (dislocation of the neck) and the brain was placed in formalin with the skull left intact to retain the shape of the brain. All brain samples were soaked in formalin for 5 days before being processed for immunohistochemistry.

### Immunohistochemistry

Fixed brains were removed from the skull and sliced into 1 mm thick coronal sections using a matrix to match the imaging slices taken during MRI. Serial sections taken from the 1 mm mouse brain slices were stained for glial fibrillary acidic protein (GFAP) [Novocastra mouse monoclonal clone GA5 supplied by Leica Microsystems, Newcastle-upon-Tyne, UK (product code: NCL-GFAP-GA5)], ionized calcium binding adaptor molecule 1 (Iba-1) [MenaPath rabbit Polyclonal antibody supplied by A. Menarini Diagnostics, Wokingham, UK (product code: MP-290-CR05)], and Aβ [Novocastra mouse monoclonal Aβ clone 6F/3D supplied by Leica Microsystems, Newcastle-upon-Tyne, UK (product code: NCL-B-Amyloid)]. For GFAP and Iba-1 staining, antigen retrieval was carried out using Epitope Retrieval Solution, pH 6 at 100 °C for 30 min; GFAP antibody was applied for 15 min at a dilution of 1:200 and Iba-1 antibody was applied for 15 min at a dilution of 1:800. For Aβ staining, sections were treated with Epitope Retrieval Solution, pH 9 at 100 °C for 30 min and 90% (v/v) formic acid for 5 min; Aβ antibody was applied for 15 min at a dilution of 1:50. Immunohistochemical staining was carried out using Bond III fully automated staining system with their Bond Polymer Refine detection system and associated reagents, supplied by Leica Microsystems, Newcastle-upon-Tyne, UK. This automated system provides high sensitivity and reproducibility in immunohistochemical staining. Digital images of the histological slides were captured using a Nikon Super Coolscan 8000 ED camera, at 100% scale and 4000 pixels/in, and using the same settings for all images.

### Quantification of T_1_ relaxation times and immunohistochemical marker area fraction

ImageJ software (Rasband, W.S., ImageJ, US National Institutes of Health, Bethesda, MD, USA, http://imagej.nih.gov/ij/, 1997–2012) [28] was used to generate T_1_ quantitative maps. For each dataset, MR images at each TR were normalised by dividing by the number of averages in its acquisition using the ImageJ maths function, and images were stacked and co-registered using the plugin, Stackreg (Thevenaz et al., Lausanne, Switzerland, 1998) [29]. T_1_ quantitative maps were generated from the stacked images using the MRI Analysis Calculator ImageJ plugin (https://imagej.nih.gov/ij/plugins/mri-analysis.html) by Karl Schmidt. Mean T_1_ relaxation times were recorded for areas that covered the lower cortex and corpus callosum. This was done for the lower cortex by placing three small ROIs (4 pixels × 4 pixels) each in the left and right hemispheres that covered the lower layers of the posterior parietal and primary somatosensory areas of the cortex (a region we call lower cortex). For the corpus callosum, two ROIs (5 pixels × 2 pixels) were placed directly below the ROIs placed in the lower cortex and over the dark area corresponding to dense myelin areas of the corpus callosum. The use of multiple ROIs, as opposed to one hand drawn selection, was chosen so to cover a large enough area whilst keeping the area and placement of ROIs consistent between all mice.

ImageJ was used to calculate the area fraction of positive staining (percentage pixels in the ROI that have been highlighted using the threshold function). The method for calculating area fraction was followed as described in the online ImageJ documentation, http://rsbweb.nih.gov/ij/docs/examples/stained-sections/index.html. Red–green–blue images were converted to 8-bit, grey scale images; a set threshold level was chosen for each of the three immunological stains that selected the areas of positive staining but did not include other areas (upper and lower threshold values: Aβ = 0–180; GFAP = 0–210; Iba-1 = 0–220). ROIs in the MR images were transferred to approximately the same anatomical regions in the histological images and increased in size by a factor of 12, equal to the difference in scale factor of the MR and histological images, and area fraction was calculated automatically.

### Statistics

All data are presented as mean ± SEM. Statistical significance of differences between experimental groups was evaluated using the SPSS software [IBM Corp. Released 2013. IBM SPSS Statistics for Windows, Version 22.0. Armonk, NY, USA: IBM Corp (http://www.IBM.com)]. Following a one-way analysis of variance (ANOVA) in a 2 × 2 factorial design, means were compared using Fisher’s unprotected least significant difference test. An additional analysis was made on the 5xFAD mice to compare the lower cortex with corpus callosum for each of the histological markers Aβ, GFAP, and Iba-1 using a paired *t* test. Data were considered to be statistically significant with *p* < 0.05. To compare Aβ load in young 5xFAD mice with that of old mice, one-way ANOVA was used to determine whether any differences between group means, i.e., Aβ load in the lower cortex and corpus callosum of 2.5-, 5-, and 11-month-old mice, were statistically significant.

## Results

### Quantification of Aβ load and gliosis in young 5xFAD mice

To understand whether the quantified T_1_ relaxation time can be used to detect early changes in the brain of young 5xFAD mice, immunohistochemistry was first applied in order to determine time points of Aβ plaque development and accompanied neuroinflammation. Although it has been reported previously that Aβ deposition and gliosis begin in some brain regions of 5xFAD mice at the age of 2 months and increase steadily afterwards [22], to date no quantitative data are available characterising Aβ load and neuroinflammation (1) in different brain regions and (2) at different ages of 5xFAD mice. To determine early changes in the brain of 5xFAD mice, we quantified Aβ deposition, astrocytosis and microgliosis in brains of 2.5- and 5-month-old 5xFAD mice and age-matched wild-type mice. Aβ staining (to determine Aβ plaque development), GFAP staining (to quantify activated astrocytes) and Iba-1 staining (to quantify activated microglial cells) were analysed in two brain regions, namely the lower cortex and the corpus callosum. We focussed specifically on these two brain regions, as they have been identified as areas with the largest differences in MRI T_1_ relaxation time upon comparison of 11-month-old wild-type and 5xFAD mice [23].

### Quantification of astrocytosis and microgliosis in young 5xFAD mice

**Fig. 1:**
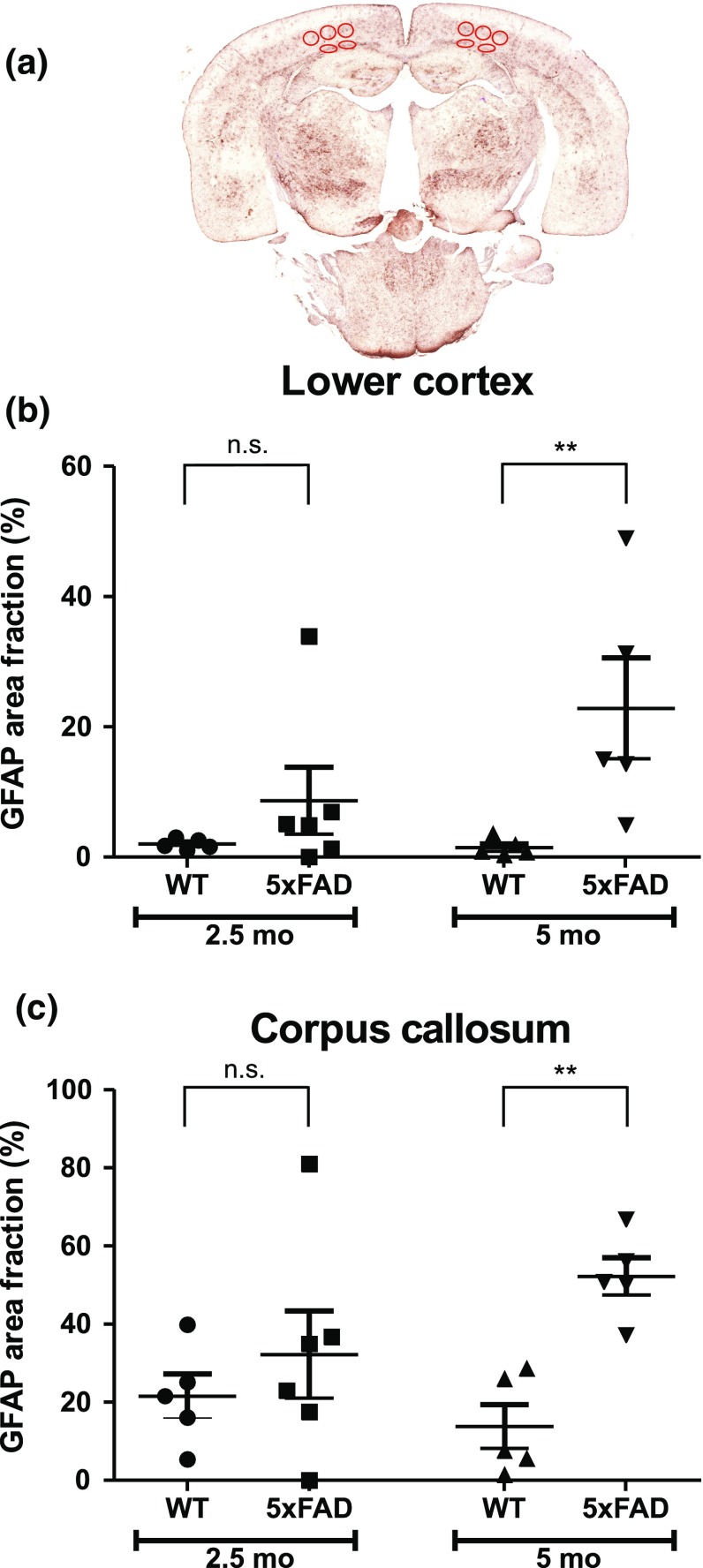
GFAP staining in wild-type (WT) and 5xFAD mice. **a** An example of a glial fibrillary acidic protein (GFAP) stained section from a 5-month-old ( 5 mo) 5xFAD mouse. *Charts* show area fraction (%) (mean ± SEM) in the lower cortex (**b**) and corpus callosum (**c**) in 2.5-month-old (2.5 mo) and 5 mo WT and 5xFAD mice. Regions of interest are shown by *red ellipses*. *n.s*. not significant; **p < 0.01

First, we quantified area fraction of GFAP staining in lower cortex and corpus callosum of 2.5- and 5-month-old wild-type and 5xFAD mice. There was a statistically significant difference between groups in the lower cortex (*p* < 0.05) and the corpus callosum (*p* < 0.05) as determined by one-way ANOVA. The Fisher’s unprotected least significant difference test was used to show which groups differed from each other. As demonstrated in Fig. 1, in both brain regions, GFAP staining was not significantly different between wild-type and 5xFAD mice at the age of 2.5 months (*p* = 0.324 for lower cortex; *p* = 0.343 for corpus callosum; WT *n* = 5, 5xFAD *n* = 6). However, at the age of 5 months, significant differences were found in the area fraction of GFAP staining between wild-type and 5xFAD mice for both regions. In comparison with wild-type mice, in 5xFAD mice, GFAP area fraction was 16-fold larger (*p* < 0.01; WT *n* = 5, 5xFAD *n* = 5) in the lower cortex, and fourfold larger (*p* < 0.01; WT *n* = 5, 5xFAD *n* = 5) in the corpus callosum. Furthermore, GFAP area fraction was significantly larger in corpus callosum than in the lower cortex of both 2.5- and 5-month-old mice (*p* < 0.05 in both cases). In comparison with 2.5-month-old 5xFAD mice, GFAP area fraction of 5-month-old 5xFAD mice was threefold increased (*p* < 0.05) in the lower cortex, but only numerically higher in the corpus callosum [twofold increased (*p* = 0.083)].

**Fig. 2:**
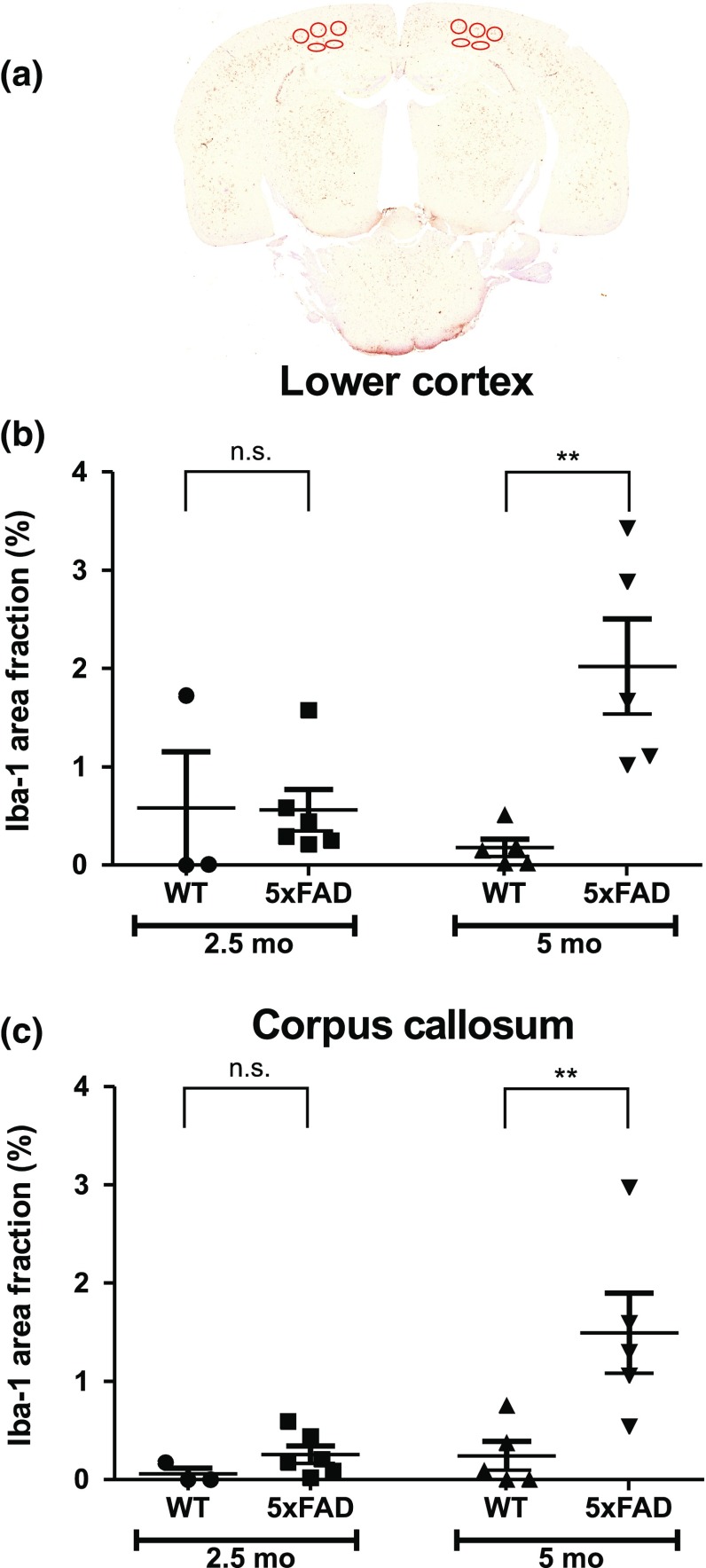
Iba-1 staining in wild-type (WT) and 5xFAD mice.** a** An example of a brain slice from a 5-month-old (5 mo) 5xFAD mouse stained with ionized calcium binding adaptor molecule 1 (Iba-1). *Charts* show area fraction (%) (mean ± SEM) in the lower cortex (**b**) and corpus callosum (**c**) in 2.5-month-old (2.5 mo) and 5 mo WT and 5xFAD mice. Regions of interest are shown by *red ellipses*. *n.s.* not significant; **p < 0.01

Furthermore, analyses of Iba-1 staining were performed to study microgliosis in young 5xFAD mice. Figure 2 shows an example of a brain slice stained with Iba-1 and summarises results of Iba-1 staining in the lower cortex and corpus callosum in wild-type and 5xFAD mice at the age of 2.5 and 5 months. Qualitatively, Iba-1 staining was similar to that obtained for GFAP staining. There was a statistically significant difference between groups in the lower cortex (*p* < 0.01) and the corpus callosum (*p* < 0.01) as determined by one-way ANOVA. Analysis of the lower cortex and of the corpus callosum did not reveal any significant differences in area fraction of Iba-1 staining between 2.5-month-old wild-type and 5xFAD mice (*p* = 0.972 for lower cortex; *p* = 0.600 for corpus callosum; WT *n* = 3, 5xFAD *n* = 6). In contrast, Iba-1 staining was much more pronounced in 5-month-old 5xFAD mice compared with 5-month-old wild-type mice. In 5-month-old 5xFAD mice, area fraction of Iba-1 staining was 12-fold higher in the lower cortex (*p* < 0.01; WT *n* = 5, 5xFAD *n* = 5) and sixfold higher in the corpus callosum (*p* < 0.01; WT *n* = 5, 5xFAD *n* = 5) in comparison with the corresponding brain areas of wild-type mice. No differences were found in Iba-1 area fraction between lower cortex and corpus callosum of either 2.5- or 5-month-old 5xFAD mice (*p* = 0.077 and *p* = 0.334, respectively). Iba-1 area fraction in the lower cortex (fourfold; *p* < 0.01) and corpus callosum (sixfold; *p* < 0.01) increased significantly in 5-month-old 5xFAD mice compared with 2.5-month-old 5xFAD mice.

### Quantification of Aβ load in young 5xFAD mice

**Fig. 3:**
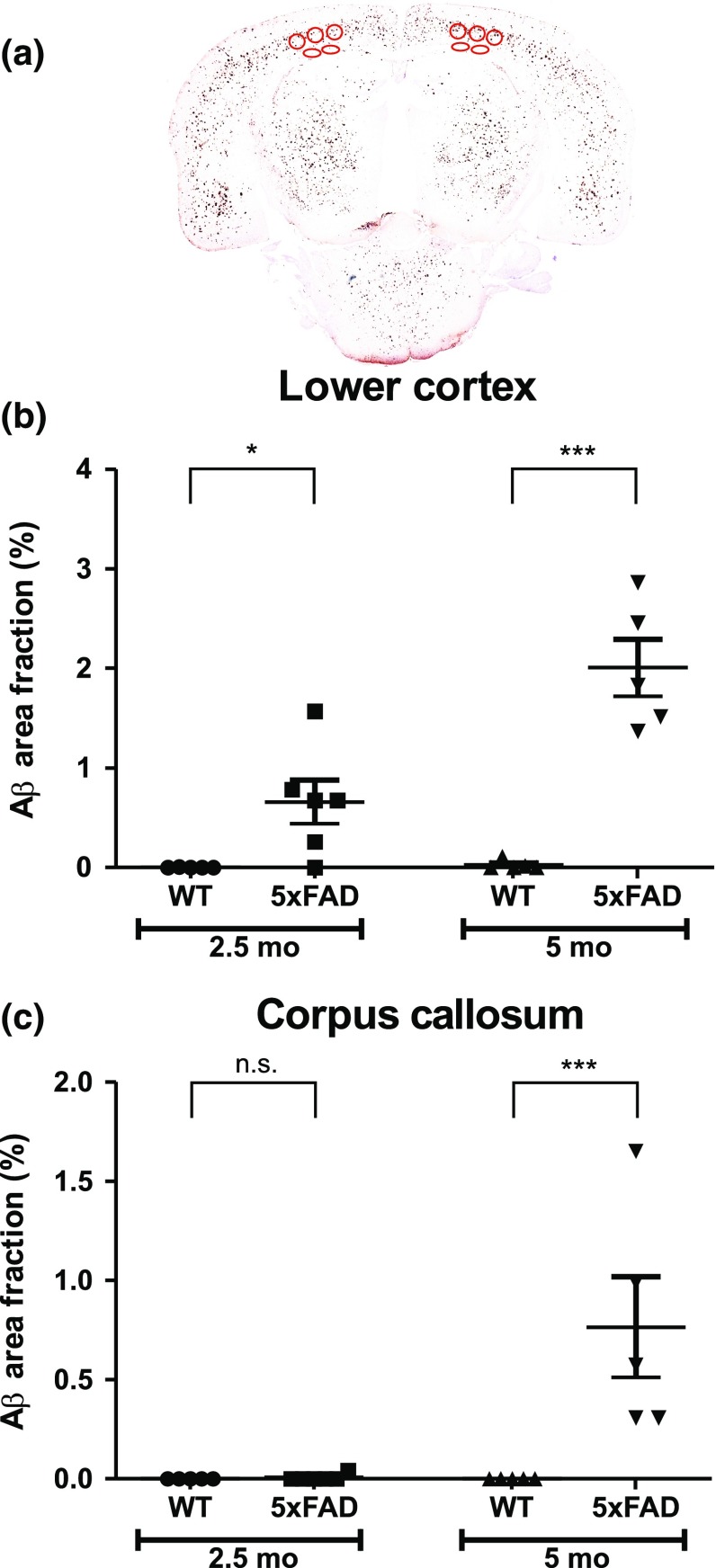
Aβ staining in wild-type (WT) and 5xFAD mice.** a** An example of an amyloid-β (Aβ) stained section from a 5-month-old (5 mo) 5xFAD mouse. Area fraction (%) (mean ± SEM) in the lower cortex (**b**) and corpus callosum (**c**) were determined in 2.5-month-old (2.5 mo) and 5 mo WT and 5xFAD mice. Regions of interest are shown by *red ellipses. n.s.*not significant; ***p < 0.001

Analysis with one-way ANOVA found statistically significant differences between groups in the lower cortex (*p* < 0.001) and corpus callosum (*p* < 0.001). No significant Aβ staining was detectable in the brain of wild-type mice of both age groups. However, in agreement with Oakley et al. [22], Aβ staining was visible in 5xFAD mice as young as 2.5-months-old. Staining was more pronounced in the lower cortex than in the corpus callosum. In 2.5-month-old 5xFAD mice, Aβ area fraction in the lower cortex was 96-fold higher (*p* < 0.05) than in the corpus callosum. Similar to observations made for 2.5-month-old mouse brains, higher amounts of Aβ were detected in the lower cortex than in the corpus callosum of 5-month-old 5xFAD mice (Fig. 3). In comparison with 5-month-old WT mice, in 5-month-old 5xFAD mice, Aβ deposition was substantially enhanced in both brain regions investigated (lower cortex: *p* < 0.001; corpus callosum: *p* < 0.001; WT *n* = 5, 5xFAD *n* = 5) (Fig. 3). Furthermore, compared with 2.5-month-old 5xFAD mice, Aβ area fraction was significantly increased in the lower cortex (threefold; *p* < 0.001) and corpus callosum (111-fold; *p* < 0.001) of 5-month-old 5xFAD mice.

### Quantification of Aβ load in old 5xFAD mice

Substantial Aβ plaque load has previously been reported for both brain regions, the lower cortex and corpus callosum, in old 5xFAD mice [22, 25]. To compare Aβ load in lower cortex and corpus callosum of young 5xFAD mice with that of old mice, we additionally quantified Aβ area fraction in brain slices obtained from 11-month-old mice using the method described above. Analysis with one-way ANOVA found statistically significant differences between groups in the lower cortex (*p* < 0.001) and corpus callosum (*p* < 0.001). In the lower cortex, Aβ area fraction of 11-month-old 5xFAD mice was 70-fold (*p* < 0.001; 2.5 mo *n* = 5, 11 mo *n* = 6) higher than that of 2.5-month-old mice and 23-fold (*p* < 0.001) higher than that of 5-month-old 5xFAD mice. Similarly, in the corpus callosum, a much higher Aβ load was found for 11-month-old mice compared with young mice. Aβ area fraction in the corpus callosum of 11-month-old 5xFAD mice was 4090-fold (*p* < 0.001; 2.5 mo *n* = 6, 11 mo *n* = 6) and 37-fold (*p* < 0.001; 5 mo *n* = 5, 11 mo *n* = 6) increased when compared with that of 5xFAD mice at the age of 2.5 months and 5 months, respectively.

### MRI T_1_ signals of young 5xFAD mice

**Fig. 4:**
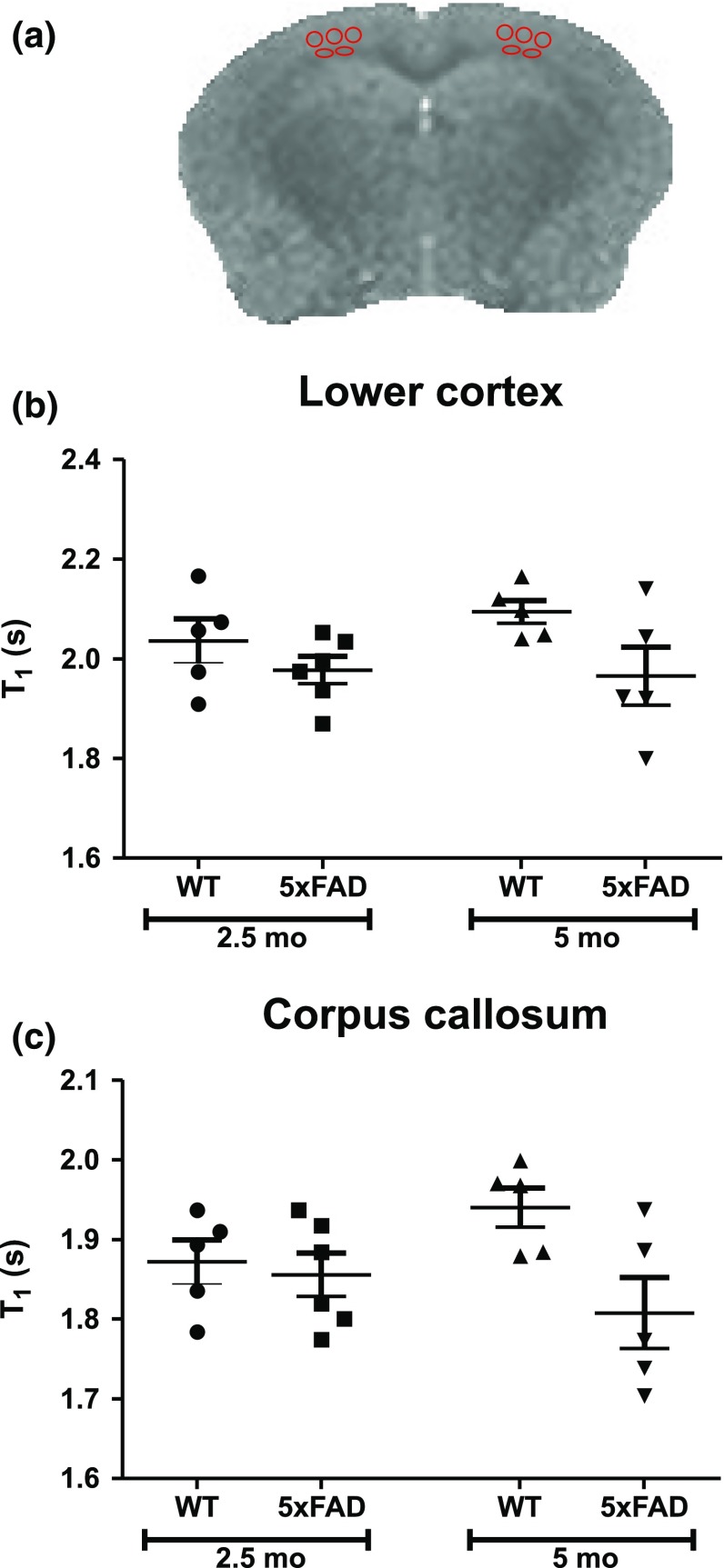
MRI T_1_ relaxation times for wild-type (WT) and 5xFAD mice.** a** An example of an MRI T_1_ quantitative map from a 5-month-old (5 mo) 5xFAD mouse. MRI T_1_ (mean ± SEM) were determined in the lower cortex (**b**) and the corpus callosum (**c**) of WT and 5xFAD mice aged 2.5 months (2.5 mo) and 5 mo. Each symbol represents data from an individual mouse. Regions of interest are shown by *red ellipses*. No statistics are shown; there was no overall significant difference between means in the one-way ANOVA, which precludes further comparison between means

We have previously reported that 11-month-old 5xFAD mice are characterised by significantly lower MRI T_1_ relaxation time compared to age-matched wild-type mice, while differences were most pronounced in the lower cortex and the corpus callosum (white matter) [25]. To test whether MRI T_1_ can be used to detect early changes in Aβ load and neuroinflammation in AD mice, we determined MRI T_1_ relaxation time in young wild-type and 5xFAD mice. No statistical significant differences in T_1_ relaxation time were found between the groups (2.5- and 5-month-old mice) as determined by one-way ANOVA, indicating that T_1_ relaxation times in the lower cortex as well as in the corpus callosum differed neither between wild-type and 5xFAD mice at both age groups nor between 2.5- and 5-month-old 5xFAD mice (Fig. 4).

## Discussion

### Histochemistry of young 5xFAD mice

In this study, we present the first quantification of amyloidosis, astrocytosis, and microgliosis in the lower cortex and the corpus callosum of young 5xFAD mice. The 5xFAD transgenic mouse was developed by Oakley et al. [22] to co-express five familial forms of Alzheimer’s disease [APP K670 N/M671L (Swedish) + I716 V (Florida) + V717I (London) and PS1 M146L + L286 V]. 5xFAD mice are characterised by the early development of Aβ plaques, neuroinflammation, neuronal loss, and memory impairment [22–24, 30, 31], all hallmarks of human Alzheimer’s disease. Therefore, these 5xFAD transgenic mice have been acknowledged as a useful model for better understanding the pathogenesis of human Alzheimer’s disease. In agreement with previous reports [22, 24], we found that first signs of amyloidosis and gliosis were detectable in the lower cortex and the corpus callosum of 2.5-month-old 5xFAD mice, although area fractions of GFAP staining and Iba-1 staining did not differ significantly from those of age-matched controls. However, significant increases in both astrocytosis and microgliosis, which occurred in parallel to significant increases in the Aβ area fraction, were found in the lower cortex, as well as in the corpus callosum of 5xFAD mice at the age of 5 months. This is the same age at which first behavioural changes, namely hippocampus-dependent memory impairments, have been observed in 5xFAD mice [22, 31]. Intriguingly, quantification of Aβ plaque load in 5-month-old 5xFAD mice compared with levels in 2.5-month-old 5xFAD mice revealed only minor increases when compared with the changes between 11- and 5-month-old 5xFAD mice. Taken together, this suggests that subtle changes in brain Aβ load and/or early development of gliosis are sufficient to cause memory deficits in an Alzheimer’s disease mouse model. Our data further point to the importance of Aβ in provoking an inflammatory response, since increases in gliosis appear to follow after the deposition of Aβ.

### MRI T_1_ relaxation times in young 5xFAD mice

As there is currently urgent need for diagnostic tools capable of identifying early changes in the brain of patients with Alzheimer’s disease, we asked whether MRI T_1_ relaxation time can be used (1) to distinguish wild-type and 5xFAD mice at an age characterised by early stages of Aβ plaque deposition and neuroinflammation and (2) to monitor disease progression in young 5xFAD mice. In a previous study, we have found that MRI T_1_ of the lower cortex and the corpus callosum were significantly different between 11-month-old 5xFAD mice and age-matched wild-type controls [25]. Brain tissue T_1_ is dependent on tissue water content [32] and is influenced by a variety of factors, which can include myelin density, axonal damage, oedema, or widening of extracellular space [33]. However, the observed early development of amyloidosis and gliosis in young 5xFAD mice was not accompanied by significant changes in MRI T_1_ relaxation time. The T_1_ differences are small between 5-month-old WT and 5xFAD mice, representing a 6–7% change in the lower cortex and corpus callosum; and there was not a significant difference between T_1_ relaxation times of 2.5- and 5-month-old 5xFAD. In a previous study [25] we found T_1_ reductions confined to specific brain regions, for example, the deep layers of the cortex; therefore, ROIs must be sized and placed appropriately to be able to capture these changes. Despite focusing our attention on the lower cortex and corpus callosum, T_1_ values were not significantly different between 5-month-old 5xFAD and WT mice. Other studies have found no T_1_ differences between AD and WT mice, for example, in PS/APP mice 16–23 months old [34], and in ArcAβ mice, which have similar T_1_ values to WT mice irrespective of age [35]. It is not known, however, if the lack of T_1_ change in those studies is due to the size and placement of ROIs or the use of different AD transgenic models.

We have suggested previously that the presence of Aβ plaques may explain differences in MRI T_1_ between 11-month-old wild-type and 5xFAD mice [25]. However, since the beginning of Aβ plaque deposition cannot be reliably detected by MRI T_1_, this suggests that only substantial Aβ load causes significant changes in MRI T_1_ (e.g., in the corpus callosum of 11-month-old mice, WT T_1_ = 1.85 s versus 5xFAD T_1_ = 1.72 s). This hypothesis is supported by our finding that the Aβ area fractions in lower cortex and corpus callosum of 11-month-old 5xFAD mice were more than 20–30 times larger than those determined for the corresponding brain regions in 5-month-old mice. In general, reports from other research laboratories of significant T_1_ differences between AD and wild-type mice were in old mice when Aβ deposition is fully established [36, 37]. Thus, an insufficient amount of Aβ in young 5xFAD mice could explain the lack of large differences in MRI T_1_ relaxation times between young wild-type and 5xFAD mice. We cannot exclude that there might have been T_1_ differences in other brain regions of the AD mouse, for example, the subiculum [36]; in this work, analysis was restricted to the lower cortex and corpus callosum, where there are easily identifiable anatomical boundaries that help to minimise the subjective nature of ROI placement, and where T_1_ changes in old mice were previously identified [25]. Nevertheless, it should be noted that there is still the possibility that differences will be found in MRI studies at high magnetic field strengths (7.0 T and above) that provide higher signal-to-noise ratio and improved spatial resolution), with T_2_ or T_2_*-weighted imaging, which enables the detection of individual Aβ plaques in the mouse [38–42], ex vivo human [43], and plaque-like pathology in vivo human brain [44], and, given the slow rate of Aβ deposition decades prior to the onset of clinical symptoms in AD [2], direct visualisation of plaques as opposed to measuring indirect effects related to Aβ deposition may provide a better early diagnostic tool. However, there are unresolved questions over the specificity of this technique, and to date, most clinical studies use the lower field strength 3 *T* MRI. Further work is needed to develop diagnostic tools capable of detecting early changes in the brain of AD patients. Previous investigations have found magnetisation transfer ratio measurements a useful means of assessing changes in tissue integrity that may involve gliosis [45–47] (an early event in AD); it would be of interest to assess other MRI methods such as MTR to give quantitative information on the AD mouse brain.

In providing in vivo evaluation of Aβ deposition, PET is closer to clinical application than MRI. PET tracers that allow direct, in vivo visualisation of Aβ are advancing in their development and may allow early detection and monitoring of treatment response. Increased Pittsburgh Compound B retention is found in AD patients compared with controls [48, 49], and florbetaben, the third amyloid imaging agent to be approved for clinical use (Amyvid [Florbetapir F 18] was approved by the FDA in 2012 [50], and Vizamyl [Flutemetamol F 18] was approved by the FDA in 2013 [51]), demonstrated high sensitivity and specificity for detecting Aβ deposits, and high negative predictive value [52]. That said, large-scale controlled trials are needed to assess further the usefulness of these techniques in people with early stage AD, who are more likely to benefit from treatment intervention targeting amyloid pathology. Furthermore, it is still important to assess other potential imaging modalities that may have use within the AD diagnostic framework as Aβ-targeted PET is not yet capable of diagnosing AD on its own.

The strength of this study is in the quantitative analysis of histological markers Aβ, GFAP, and Iba-1 in young 5xFAD mice, which takes into account all samples and allows a more objective analysis compared with simply describing changes seen by the eye. The limitations of this study relate to the relatively low MR imaging resolution (0.23 × 0.23 × 1 mm). Despite there being no compelling evidence to suggest the mean T_1_ values differ between young 5xFAD and WT mice, we cannot reach a strong conclusion; repeated experiments with larger samples would be needed; however, given the very small differences in T_1_ between 5-month-old 5xFAD and WT mice this would not be recommended based on the results of this study. The small size of the mouse brain poses challenges for high-resolution MR imaging. The use of high field MRI systems can deliver higher signal-to-noise ratio than what was achievable on the 4.7 T system used in this study; we cannot neglect the impact improved image quality and higher imaging resolution would have on improving detection. Another important limitation is the discrepancy between mouse models and AD brains, which may arise from differences in tissue composition and structure, for example, Aβ load, tissue-water content, iron accumulation, and loss of fibre tracts; therefore, the mouse model does not fully recapitulate all the features of AD. For example, important differences in the characteristics of plaques in human AD and APP/PS1 mice may provide different mechanisms of MR contrast changes: plaques in APP/PS1 mice contain less iron, but are more densely packed compared with human AD samples [53]. Using voxel-based quantification, Su et al. found that AD was characterised by reduced T_1_ values in certain brain regions, including bilateral temporal and parietal lobes in cross-sectional comparison, and an increase in T_1_ in the right caudate, bilateral hippocampus, parahippocampus, and thalamus in longitudinal comparison [20]. Increased T_1_ values in white matter of AD patients has been reported [54], and taken together this demonstrates the complex nature of T_1_ changes in the human AD brain. Caution is needed when translating results from animal to human, and it would be important for future studies to assess more than one transgenic mouse model. The difference between changes in T_1_ values in mouse models and human AD may also arise from the relatively low specificity of the measurement. Since brain tissue T_1_ is dependent on a variety of factors, the effect of ongoing disease on tissue composition may confound using a simple T_1_ relaxation time as a measure of treatment response or disease progression.

## Conclusion

Our data do not provide sufficient evidence that the mean T_1_ values differ between WT and 5xFAD mice at young ages (2.5 and 5 months) despite the transgenic mice having pronounced pathology by the age of 5 months compared with age-matched WT mice.

## Abbreviations

5xFAD: Five familial Alzheimer’s disease mutations
Aβ: Amyloid-β
AD: Alzheimer’s disease
FOV: Field of view
GFAP: Glial fibrillary acidic protein
Iba-1: Ionized calcium binding adaptor molecule 1
PET: Positron emission tomography
Mo: Month
MRI: Magnetic resonance imaging
ROI: Region of interest
TE: Echo time
TR: Repetition time
WT: Wild-type
